# Numerical Simulation on Convection and Thermal Radiation of Casson Fluid in an Enclosure with Entropy Generation

**DOI:** 10.3390/e22020229

**Published:** 2020-02-18

**Authors:** A. K. Alzahrani, S. Sivasankaran, M. Bhuvaneswari

**Affiliations:** 1 Department of Mathematics, King Abdulaziz University, Jeddah 21589, Saudi Arabia; akalzahrani@kau.edu.sa; 2 Department of Mathematics, Kongunadu Polytechnic College, D.Gudalur, Dindigul, Tamilnadu 624620, India; msubhuvana@yahoo.com

**Keywords:** aspect ratio, entropy, convection, thermal radiation, Casson fluid, cavity

## Abstract

The goal of the current numerical simulation is to explore the impact of aspect ratio, thermal radiation, and entropy generation on buoyant induced convection in a rectangular box filled with Casson fluid. The vertical boundaries of the box are maintained with different constant thermal distribution. Thermal insulation is executed on horizontal boundaries. The solution is obtained by a finite volume-based iterative method. The results are explored over a range of radiation parameter, Casson fluid parameter, aspect ratio, and Grashof number. The impact of entropy generation is also examined in detail. Thermal stratification occurs for greater values of Casson liquid parameters in the presence of radiation. The kinetic energy grows on rising the values of Casson liquid and radiation parameters. The thermal energy transport declines on growing the values of radiation parameter and it enhances on rising the Casson fluid parameter.

## 1. Introduction

Since convective flow with heat energy transfer plays a major role in numerous places, research on this topic has been executed over the last few decades [1,2,3,4,5]. However, the research on convective flow of Casson fluids is not active up to the level of current scientific and technological requirements. Therefore, we investigate the energy transport and flow of Casson fluid under different physical environments due to its applications in electronics manufacturing, printing, textile printing, semi-solid casting, etc. [6,7,8]. The Casson liquid is one kind of non-Newtonian liquid and it is a shear thinning (pseudo-plastic) liquid which behaves like an elastic solid [5]. Pop and Sheremet [9] explored the convective stream of Casson fluid in an enclosed space in the occurrence of radiation and viscous dissipation. They established that the Casson parameter favors energy transferal augmentation and stream intensification. Sheremet and Pop [10] explored the convective flow with thermal radiation of a visco-elastic fluid in a square domain. They attained the energy reduction while increasing the elastic number. Srinivas et al. [11] inspected the influence of thermal radiation and chemical reaction on hydromagnetic pulsating Casson liquid flow in a porous channel. The study on buoyant convective stream in the existence of thermal radiation has been enriched in research due to applications in various fields [12,13,14,15]. Mansour et al. [16] observed the impact of thermal radiation on free convection in a wavy box. They established that averaged Nusselt number rises by raising the radiation values. Mahapatra et al. [17] surveyed the combined radiation and convection in a porous box in the occurrence of heat generation. Miroshnichenko et al. [18] inspected the combined radiation and convective motion in enclosures with a heat-generating component.

The entropy generation is the amount of entropy which is produced in any irreversible processes, such as heat/mass transfer, viscous fluid flow, chemical reactions, and Joule heating [19,20]. Every process in nature provides a positive or zero value of entropy generation rate. It is an essential aspect of the 2nd law of thermodynamics [21,22]. The study of entropy generation in closed domain has been analyzed in several studies [23,24,25]. Ahmed et al. [26] analyzed the entropy production due to doubly diffusive convection of Casson liquids in the occurrence of slip boundaries and chemical reactions. They observed that the Casson parameter diminishes the entropy production. Hajji et al. [27] explored the entropy production due to combined free convection and radiative transfer in an inclined box. They concluded that entropy generation rises by increasing wall emissivity due to surface radiation. Daniel et al. [28] explored mixed magneto-convection of chemically reacting nanoliquid with the combined influence of thermal radiation and viscous dissipation. The entropy generation on doubly diffusive convection of Carreau fluid with Dufour and Soret effects and viscous dissipation in a box was investigated by Kefayati and Tang [29]. They found that the growth of Dufour and Soret parameters enhance the entropy production. In another study, Kefayati [30] explored the entropy production due to doubly diffusive convection of Bingham fluid in a tilting box. Chamkha et al. [31] investigated the entropy propagation on MHD convection of nanoliquid in a porous driven box in the existence of partial slip under various heat sinks and sources. 

It is understood from the literature that there are several studies involving the convective flow with thermal radiation of Newtonian fluids. In addition, a few studies dealt with the convective stream of Casson liquid in the absence of radiation. This is motivated to initiate the present study to inspect the interaction of thermal radiation and convection of Casson fluid inside a rectangular box. Hence, the numerical model on convective current of Casson liquid in a box in the occurrence of thermal radiation with entropy generation is explored here and it has not been described in the literature.

## 2. Mathematical Model

**Figure 1:**
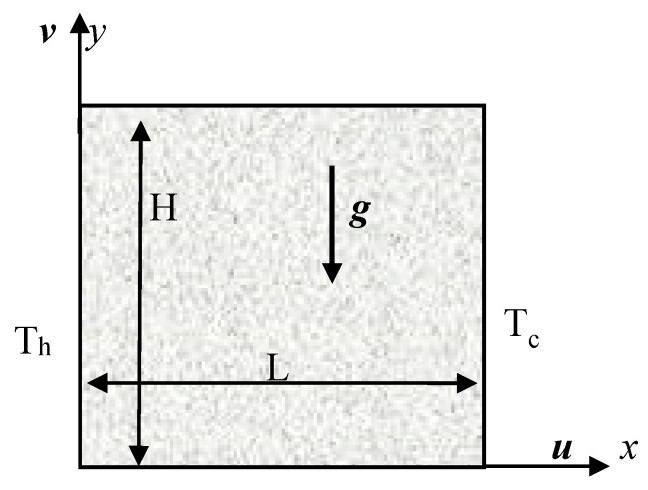
Physical domain.

Buoyant convective laminar incompressible flow in a 2-dimensional box of height *H* and width L filled with Casson liquid is considered in the current study, as given in Figure 1. The left wall has the constant higher temperature $\left(T(y)=T_{h}\right)$ and right wall has constant lower temperature $\left(T(y)=T_{c}\right)$. The thermal insulation is imposed on horizontal walls. The gravity acts in the negative y-direction. The thermal radiation is taken into account. The medium is optically thick. The thermal properties of the liquid are kept invariable except the density in the buoyancy term (Boussinesq approximation). The density is defined as $\rho =\rho_{0}[1-\beta_{T}(T-T_{c})]$, where *β_T_* is thermal expansion coefficient. No viscous dissipation or Joule heating are counted.

The rheological model of Casson fluid is derived as below [9,10]:

(1) $$\tau_{ij}=\{\begin{array}{ll} 2\left(\mu_{B}+\frac{P_{y}}{\sqrt{2\pi }}\right)e_{ij}, & \pi >\pi_{C}\\ 2\left(\mu_{B}+\frac{P_{y}}{\sqrt{2\pi_{C}}}\right)e_{ij}, & \pi <\pi_{C} \end{array}$$

where $\pi =e_{ij}.e_{ij}$ and $e_{ij}$ are (*i*, *j*)th component of the deformation rate, $\pi_{c}$ is a critical rate of the product of deformation rate, $\mu_{B}$ and $P_{y}$ is dynamic viscosity, yield stress of liquid. The Casson liquid satisfies the Boussinesq approximation. According to aforementioned assumptions, the principal equations are

(2) $$\frac{\partial u}{\;\partial x}+\frac{\partial v}{\partial y}=0$$

(3) $$\frac{\partial u}{\partial t}+\left[u\frac{\partial u}{\partial x}+v\frac{\partial u}{\partial y}\right]=-\frac{1}{\rho_{0}}\frac{\partial p}{\partial x}+\nu \left(1+\frac{1}{\beta }\right)\left[\frac{\partial^{2}u}{\partial x^{2}}+\frac{\partial^{2}u}{\partial y^{2}}\right]$$

(4) $$\frac{\partial v}{\partial t}+\left[u\frac{\partial v}{\partial x}+v\frac{\partial v}{\partial y}\right]=-\frac{1}{\rho_{0}}\frac{\partial p}{\partial y}+\nu \left(1+\frac{1}{\beta }\right)\left[\frac{\partial^{2}v}{\partial x^{2}}+\frac{\partial^{2}v}{\partial y^{2}}\right]\;+g\beta \left(T-T_{c}\right)$$

(5) $$\frac{\partial T}{\partial t}+u\frac{\partial T}{\partial x}+v\frac{\partial T}{\partial y}=\frac{k}{\rho_{0}c_{p}}\left[\frac{\partial^{2}T}{\partial x^{2}}+\frac{\partial^{2}T}{\partial y^{2}}\right]-\frac{1}{\rho_{0}c_{p}}\left[\frac{\partial q_{r}}{\partial x}+\frac{\partial q_{r}}{\partial y}\right]$$

where β is the Casson liquid parameter. The heat flux due to radiation along *x* and *y* directions are given by

(6) $$q_{r}{}_{x}=\frac{-4\sigma^{*}}{3K^{\prime }}\frac{\partial T^{4}}{\partial x}\;\mathrm{and}\;q_{r}{}_{y}=\frac{-4\sigma^{*}}{3K^{\prime }}\frac{\partial T^{4}}{\partial y}$$

Using Rosseland approximation for radiation, the temperature differences within the stream are too small. Expanding $T^{4}$ about $T_{c}$ using Taylor series and approximating it. Finally, we get

$$T^{4}≅4T_{c}{}^{3}T-3T_{c}{}^{4}$$

Therefore, heat flux due to radiation along x and y directions (Equation (6)) reduces to

(7) $$q_{r}{}_{x}=\frac{-16\sigma^{*}T_{c}{}^{3}}{3K^{\prime }}\frac{\partial T}{\partial x}\;\mathrm{and}\;\;q_{r}{}_{y}=\frac{-16\sigma^{*}T_{c}{}^{3}}{3K^{\prime }}\frac{\partial T}{\partial y}$$

Substituting Equation (7) into Equation (5), we get

(8) $$\frac{\partial T}{\partial t}+u\frac{\partial T}{\partial x}+v\frac{\partial T}{\partial y}=\frac{k}{\rho_{0}c_{p}}\left[\frac{\partial^{2}T}{\partial x^{2}}+\frac{\partial^{2}T}{\partial y^{2}}\right]+\frac{1}{\rho_{0}c_{p}}\frac{16\sigma^{*}T_{c}{}^{3}}{3K^{\prime }}\left[\frac{\partial^{2}T}{\partial x^{2}}+\frac{\partial^{2}T}{\partial y^{2}}\right]$$

The relevant initial and boundary settings are

(9) $$\begin{array}{cc} t=0:\;u=v=0,\;T=T_{0} & 0\leq x\leq L,\;0\leq y\leq H\\ t>0:\;u=v=0, & x=0\&L,y=0\&H\\ T\left(y\right)=T_{h} & x=0\\ T\left(y\right)=T_{c} & x=L\\ T_{y}=0 & y=0\&L \end{array}$$

The dimensionless form of governing model is done by the following variables:

(10) $$X=\frac{x}{L},\;Y=\frac{y}{L},\;U=\frac{uL}{\nu },\;V=\frac{vL}{\nu },\;\theta =\frac{T-T_{c}}{\left(T_{h}-T_{c}\right)},\;\tau =\frac{t\nu }{L^{2}},\;\mathrm{and}\;P=\frac{pL^{2}}{\rho_{0}\nu^{2}}$$

The non-dimensional equations are

(11) $$\frac{\partial U}{\partial X}+\frac{\partial V}{\partial Y}=0$$

(12) $$\frac{\partial U}{\partial \tau }+\left[U\frac{\partial U}{\partial X}+V\frac{\partial U}{\partial Y}\right]\;=\;-\frac{\partial P}{\partial X}+\;\left(1+\frac{1}{\beta }\right)\nabla^{2}U$$

(13) $$\frac{\partial V}{\partial \tau }+\left[U\frac{\partial V}{\partial X}+V\frac{\partial V}{\partial Y}\right]\;=\;-\frac{\partial P}{\partial Y}+\;\left(1+\frac{1}{\beta }\right)\nabla^{2}V+Gr\;\theta$$

(14) $$\frac{\partial \theta }{\partial \tau }+U\frac{\partial \theta }{\partial X}+V\frac{\partial \theta }{\partial Y}=\frac{1}{Pr}\left(1+\frac{4}{3}Rd\right)\left[\frac{\partial^{2}\theta }{\partial X^{2}}+\frac{\partial^{2}\theta }{\partial Y^{2}}\right]$$

The non-dimensional parameters in the equations are $Ar=\frac{H}{L}$ aspect ratio, $Gr=\frac{g\beta \left(T_{h}-T_{c}\right)L^{3}}{\nu^{2}}$ the Grashof number, $Pr=\frac{\nu }{\alpha }\;\left(=10\right)$, the Prandtl number, and the radiation parameter, $Rd=\frac{4\sigma^{*}T_{0}{}^{3}}{kK^{\prime }}$.

The initial and border settings (in dimensionless form) are

(15) $$\begin{array}{cc} \tau =0:\;V=U=0\;\theta =0 & 0\leq X\leq 1,\;0\leq Y\leq Ar,\\ \tau >0:\;V=U=0\;\theta_{Y}=0 & Y=0\;\&\;Ar,\\ V=U=0\;\;\theta =1 & X=0,\\ V=U=0\;\theta =0 & X=1. \end{array}$$

The thermal energy transferal rate across the box is an essential factor in the heat removal applications. The Nusselt number is the ratio of convection to conduction thermal transport across the box. The local Nusselt number along the left-wall of the box is derived as

$$Nu=\frac{q_{w}L}{k\left(\Delta T\right)}\;\mathrm{where}\;q_{w}=-\left(k\frac{\partial T}{\partial x}+\frac{16\sigma^{*}T_{c}{}^{3}}{3K^{\prime }}\frac{\partial T}{\partial x}\right).$$

The non-dimensional form of local Nusselt number is derived as follows:

$$Nu=\left(1+\frac{4}{3}Rd\right){\left(-\frac{\partial \theta }{\partial X}\right)}_{X=0}.$$

The total heat energy transfer across the box is computed by the averaged Nusselt number, which is derived as follows:

$$\bar{Nu}=\frac{1}{Ar}\int_{0}^{Ar}NudY.$$

The drag force on the surface is explored by the skin friction and it is defined as $Cf_{loc}=\frac{\partial u}{\partial x}$. The averaged skin friction factor at left-wall is estimated by integrating $Cf_{loc}$ over the length of the left-wall as follows:

$$Cf_{y}=\frac{1}{Ar}\int_{0}^{Ar}Cf_{loc}dY.$$

## 3. Entropy Generation Analysis

The buoyant convection in a closed box presents significant possibilities for thermal industrial applications. Though, the usage of entropy generation analysis assists to detect the optimal conditions for real-world applications. Since the entropy production is due to the irreversible process of heat transfer and viscosity effects, entropy production can be calculated from the known velocity and thermal fields. 

The local entropy generation per unit area can be expressed by two quantities. That is, the local entropy generation subject to heat transference and due to fluid friction [24,25,26,27,28].

(16) $$S_{heat}=\frac{k}{T_{c}^{2}}\left[{\left(\frac{\partial T}{\partial x}\right)}^{2}+{\left(\frac{\partial T}{\partial y}\right)}^{2}\right]$$

(17) $$S_{fluid}=\left(\frac{\mu }{T_{c}}\right)\left(1+\frac{1}{\beta }\right)\left\{2\left[{\left(\frac{\partial u}{\partial x}\right)}^{2}+{\left(\frac{\partial v}{\partial y}\right)}^{2}\right]+{\left(\frac{\partial u}{\partial y}+\frac{\partial v}{\partial x}\right)}^{2}\right\}$$

That is, the total local entropy production is sum of the above two quantities.

(18) $$S_{Gen}=\frac{k}{T_{c}^{2}}\left[{\left(\frac{\partial T}{\partial x}\right)}^{2}+{\left(\frac{\partial T}{\partial y}\right)}^{2}\right]+\left(\frac{\mu }{T_{c}}\right)\left(1+\frac{1}{\beta }\right)\left\{2\left[{\left(\frac{\partial u}{\partial x}\right)}^{2}+{\left(\frac{\partial v}{\partial y}\right)}^{2}\right]+{\left(\frac{\partial u}{\partial y}+\frac{\partial v}{\partial x}\right)}^{2}\right\}$$

The dimensionless form for entropy generation is obtained in a usual way by using Equation (10)

$$S_{total}=S_{heat}^{*}+S_{fluid}^{*}$$

(19) $$S_{heat}^{*}={\left(\frac{\partial \theta }{\partial X}\right)}^{2}+{\left(\frac{\partial \theta }{\partial Y}\right)}^{2}$$

(20) $$S_{fluid}^{*}=\phi_{2}\left(1+\frac{1}{\beta }\right)\left\{2\left[{\left(\frac{\partial U}{\partial X}\right)}^{2}+{\left(\frac{\partial V}{\partial Y}\right)}^{2}\right]+{\left(\frac{\partial U}{\partial Y}+\frac{\partial V}{\partial X}\right)}^{2}\right\}$$

The overall entropy generation is attained by integrating the local entropy production over the box.

(21) $$SG_{total}=\int_{V}^{}S_{total}\left(X,Y\right)dA$$

The local Bejan number specifies the strength of the entropy production subject to heat transference irreversibility. It is defined as

(22) $$Be_{loc}=\frac{S_{heat}^{*}}{S_{total}}$$

For any point inside the box, when $Be_{loc}>\frac{1}{2}$, the heat transfer irreversibility is dominating. When $Be_{loc}<\frac{1}{2}$, the liquid friction irreversibility dominates. If $Be_{loc}=\frac{1}{2}$, the viscous and thermal irreversibilities are equal. The averaged Bejan number is used to define the relative significance of the heat energy transfer irreversibility for the entire cavity.

(23) $$Be=\frac{\int_{A}^{}Be_{loc}\left(X,Y\right)dA}{\int_{A}^{}dA}$$

The computer program built-up for the flow and thermal fields is extended to calculate the entropy generation inside the box. 

## 4. Cup Mixing Temperature and RMSD 

To explore the thermal mixing in the enclosed box, cup mixing temperature is defined. The velocity-weighted averaged temperature is more suitable for convective flow than spatial average temperature. The cup mixing temperature (T_cup_) and area averaged temperature (T_avg_) are given as [32]

(24) $$T_{Cup}=\frac{\iint \hat{V}\left(X,Y\right)\;\theta \left(X,Y\right)dXdY}{\iint \hat{V}\left(X,Y\right)dXdY}$$

where $\hat{V}\left(X,Y\right)=\sqrt{U^{2}+V^{2}}$ and

(25) $$T_{avg}=\frac{\iint \theta \left(X,Y\right)dXdY}{\iint dXdY}$$

Also, root mean square deviation (RMSD) is derived to measure the degree of temperature regularity in all cases. The RMSDs are derived based on cup mixing temperature and area averaged temperature as follows:

(26) $$RMSD_{T_{cup}}=\sqrt{\frac{\sum_{i=1}^{N}{\left(\theta_{i}-T_{Cup}\right)}^{2}}{N}}$$

(27) $$RMSD_{T_{avg}}=\sqrt{\frac{\sum_{i=1}^{N}{\left(\theta_{i}-T_{avg}\right)}^{2}}{N}}$$

The higher values of RMSD indicate lower temperature uniformity inside the box and vice-versa. Also, RMSD should not exceed 1, because the dimensionless temperature varies between 0 and 1. The above parameters are calculated using the obtained values of thermal and flow fields in the same computer program. 

## 5. Numerical Technique and Validation 

The non-dimensional model Equations (11)–(14) subject to the boundary settings (Equation (15)), are solved by the control volume method [2] using a 122 × 122 non-uniform grid. The discretization of diffusive and convective terms are taken by central difference and QUICK schemes. The iterative process is executed by using a tri-diagonal matrix. The convergence condition of the iteration is kept as 10 ^−6^. The justification of current computer program is tested against the existing results for buoyant convection in a box [33,34], see Table 1. An agreement between the results provides assurance in the accurateness of the current code to explore the problem.

## 6. Results and Discussion

The numerical investigation is done to conclude the influence of the thermal radiation on natural convection flow in a Casson fluid saturated porous square box. The controlling parameters for this investigation are the Grashof number ($Gr=10^{4}\&10^{6}$), the aspect ratio (0.25≤Ar≤5), the Casson fluid parameter (0.01≤β≤1), and the radiation parameter (0≤Rd≤10). The calculations have been computed for diverse combinations of above said parameters to analyze various effects. 

**Figure 2:**
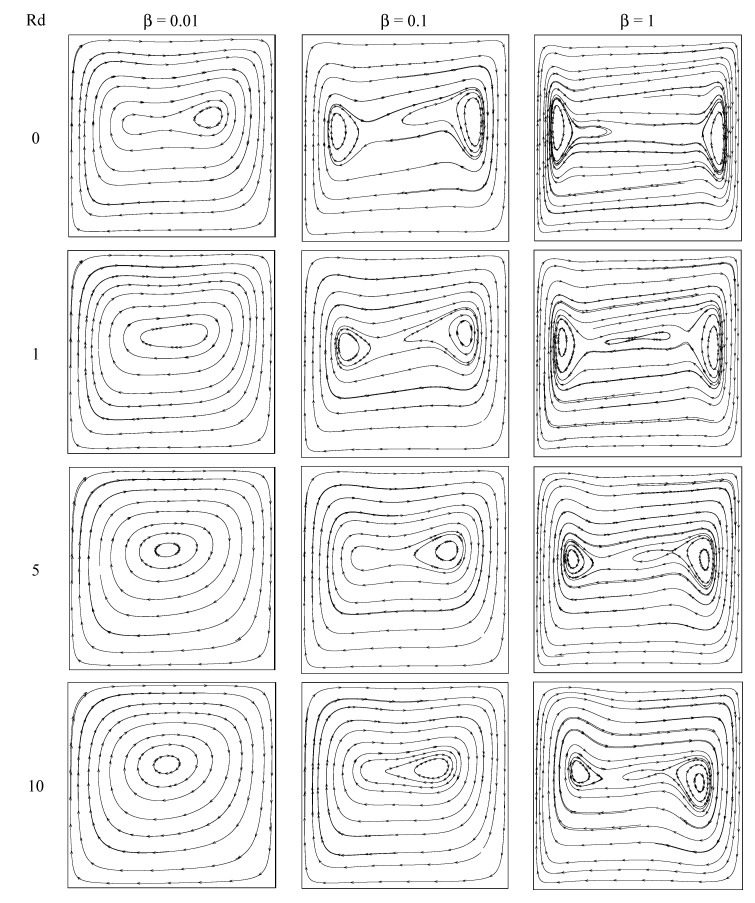
Streamlines for diverse values of radiation and Casson fluid parameters with Gr = 10^6^, Ar = 1.

**Figure 3:**
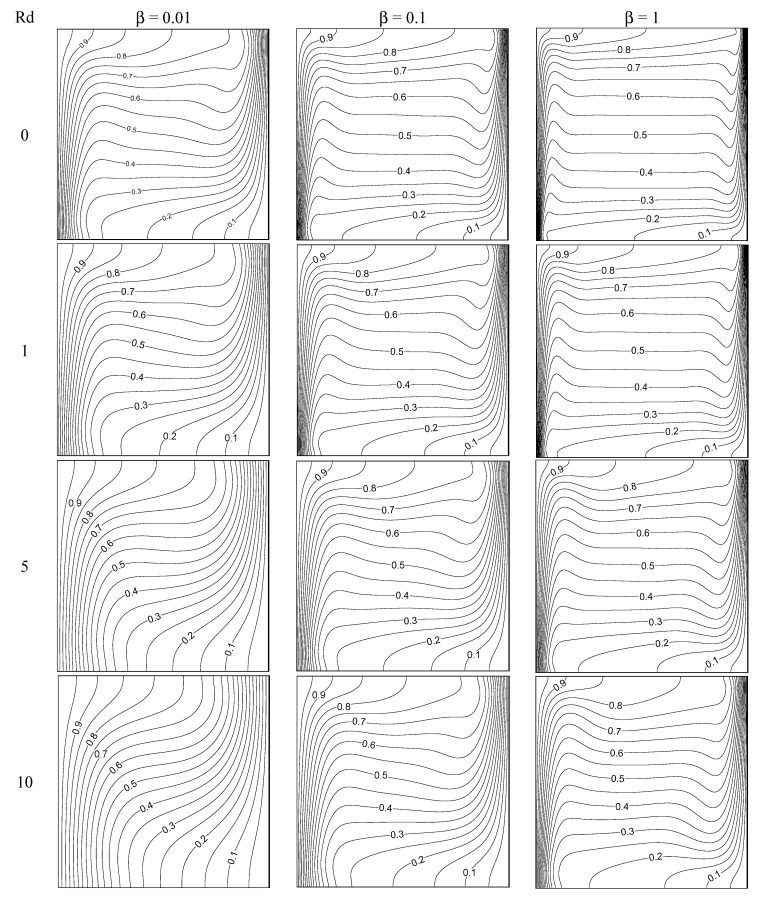
Isotherms for diverse values of radiation and Casson fluid parameters with Gr = 10^6^, Ar = 1.

The stream pattern for various values of Casson liquid parameter and thermal radiation parameter with $Gr=10^{6},Ar=1$ is displayed in Figure 2. A clockwise circling eddy occupies the whole box for all values of the considered parameters. The core region of the eddy is elongated horizontally and lies in the middle of the box in the nonappearance of thermal radiation for β = 0.01. On raising the thermal radiation intensity, the shape of the core region turns from elliptical shape to circular shape. However, this pattern is not found on raising the values of Casson fluid parameter to β = 0.1 to β = 1. That is, the viscous diffusion dominates and it slowed down the flow. The core region is divided into two parts for all values of thermal radiation parameter when β = 1 case. However, the similar trend exists for weak thermal radiation case when β = 0.1 and it disappears on strong thermal radiation case. That is, the stream speed is reduced on growing the values of *β*. Figure 3 displays the thermal distributions inside the box for several values of *β* and *Rd* with $Gr=10^{6},\;\;Ar=1$. The thermal layers at boundary are molded along the isothermal walls for all given values of Casson liquid parameter in the nonappearance of radiation parameter. The thermal boundary layers are feeble in the presence of radiation parameter and they disappear for strong values of radiation parameter (*Rd* = 10). The vertical temperature stratification is found for all *Rd* values when β = 1. The thermal layers near boundary are stronger on rising the values of *β*.

**Figure 4:**
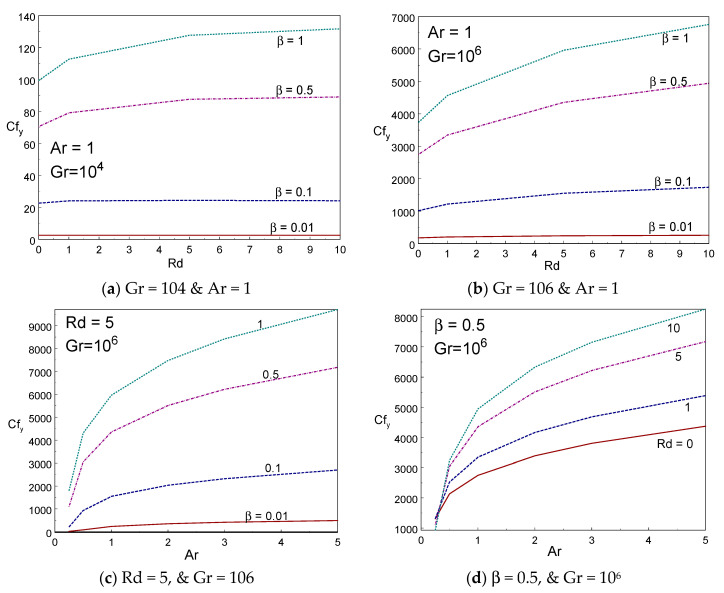
Skin friction vs. radiation (**a**,**b**) and Ar (**c**,**d**) for different values of β and Rd.

**Figure 5:**
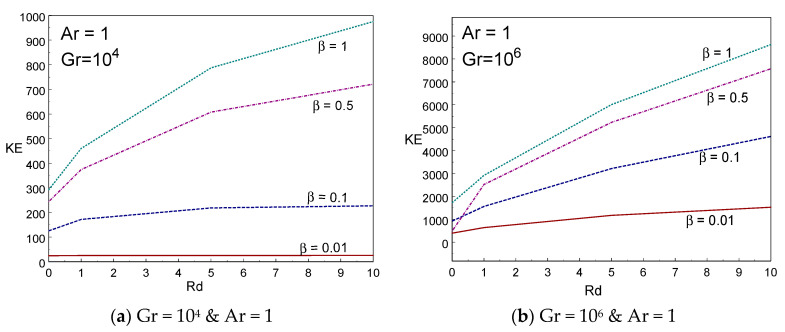

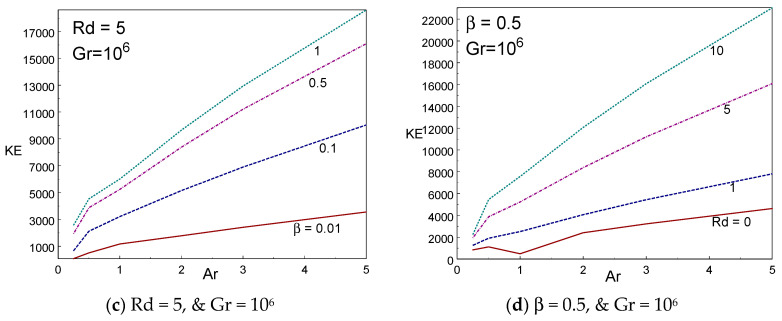
Kinetic energy vs. radiation (**a**,**b**) and Ar (**c**,**d**) for different values of β and Rd.

Figure 4a–d demonstrates the impact of skin friction ($Cf_{y}$) of the liquid for various values of thermal radiation, aspect ratio, and Casson fluid parameters. It is visibly understood from Figure 4a,b that skin friction rises on growing the values of Casson fluid parameter. Since the liquid friction inside the liquid rises on growing the Casson liquid parameter, it produces more friction between the liquid molecules and it results in resistant force being exerted on moving liquid due to drag caused by the viscosity of liquids. Comparing Figure 4a and Figure 4b, the skin friction rises in large amount by increasing Gr. The influence of aspect ratio on skin friction is clearly demonstrated in Figure 4c,d. The drag force rises while the aspect ratio of the box rises. The friction also grows when rising the values of radiation parameter. Figure 5a–d demonstrates the impact of kinematic energy (KE) of the liquid for various values of thermal radiation, aspect ratio, and Casson fluid parameters. It is visibly understood from Figure 5a,b that the kinematic energy rises on growing the values of Casson liquid parameter. However, the increment of KE augments on rising the values of the Gr number. When rising the radiation parameter to Rd = 1, 5, and 10, the KE of the liquid particle increases.

**Figure 6:**
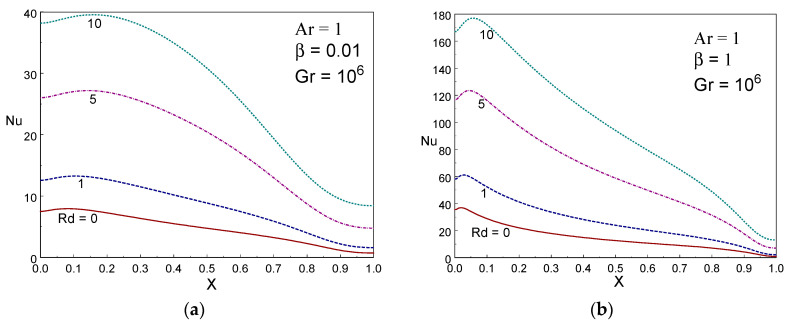
Local Nusselt number for various values of Rd with β = 0.01, and 1, Gr = 10^6^, Ar = 1.

**Figure 7:**
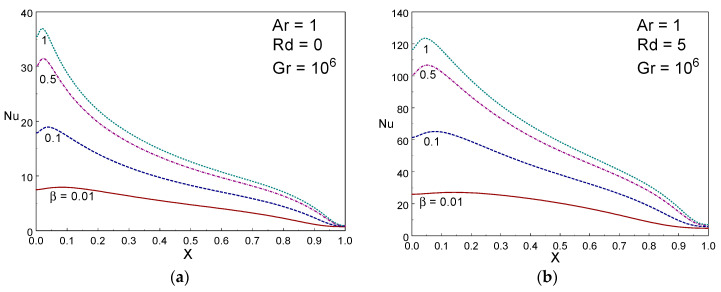
Local Nusselt number for different values of Casson liquid parameter with Rd = 0 and 5 Gr = 10^6^, Ar = 1.

Figure 6 indicates the local Nusselt number for various radiation parameter with β = 0.1 and 1, $Gr=10^{6},$ and Ar=1. The shapes of local Nusselt number curve visibly establish the impact of rate of the local thermal transport from the wall. The maximum local energy transfer is attained near the upper portion of the left wall. It is spotted that the local energy transference enhances on rising the values of radiation parameter (*Rd*). The local energy transport raises first and then declines along the wall for a given *Rd* value. Figure 7 depicts the impact of local thermal energy transport for several values of Casson liquid parameter (β) with Rd=0 & 5, $Gr=10^{6}$, and Ar = 1. The local energy transfer increases first along the height of warm wall up to *y*=0.05 and then it drops along y direction. It is also achieved the highest local energy transfer at the topmost area of the hot wall. The local energy transfer increases with β.

**Figure 8:**
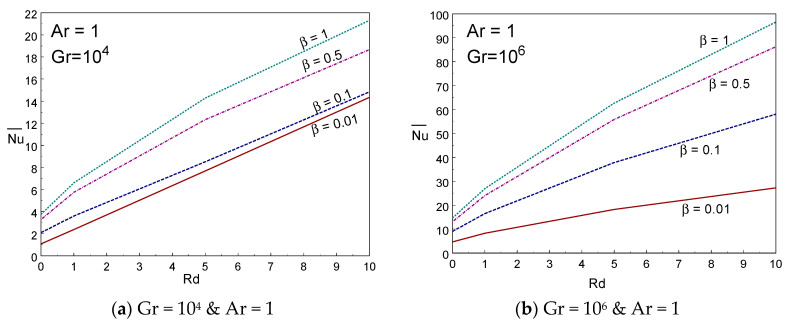

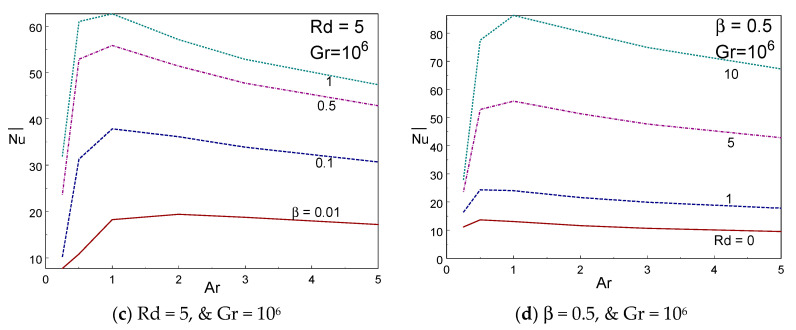
Averaged Nusselt number vs. radiation (**a**,**b**) and Ar (**c**,**d**) for various values of β and Rd.

**Figure 9:**
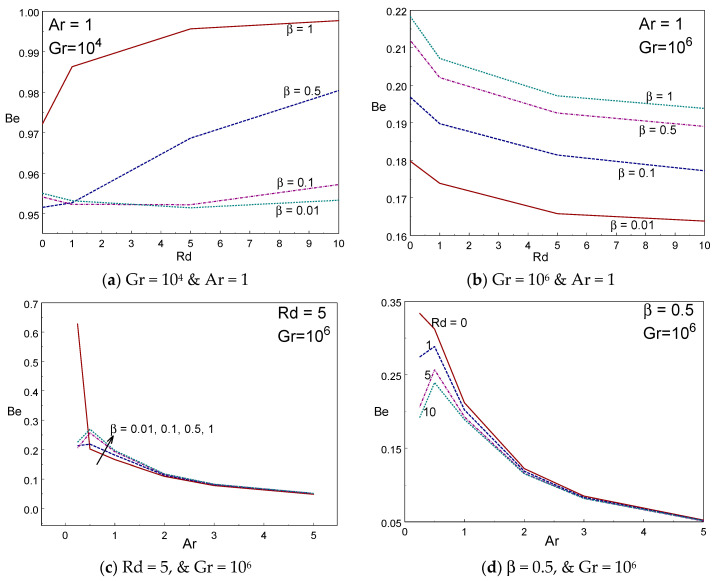
Bejan number vs. radiation (**a**,**b**) and Ar (**c**,**d**) for different values of β and Rd.

To determine the influence of thermal energy transference across the domain, the mean Nusselt number is graphed in Figure 8a–d against Rd and Ar for several values of radiation and Casson liquid parameters. It is clearly seen from these figures that the thermal energy transmission enhances on rising the values of Rd. The averaged Nusselt number rises rapidly for lower values of Ar and then falls slightly for higher values of Ar for a given Rd and β. It is also acquired that the mean energy transfer enhances on rising the values of Casson liquid parameter for all Rd values. The mean energy transport enhances on growing the values of radiation parameter. At Rd = 0, the averaged Nusselt number is almost constant when changing the values of Ar. Figure 9a–d depicted the influence of Bejan number for diverse values of Casson fluid and radiation parameters and aspect ratio. The Bejan number behaves nonlinearly with Rd for lower values of Gr = 10^4^. However, the Bejan number declines when raising the values of Rd for Gr = 10^6^. There is no difference on Bejan number when changing the values of Casson fluid parameter at high aspect ratios (Ar ≥ 3).

**Figure 10:**
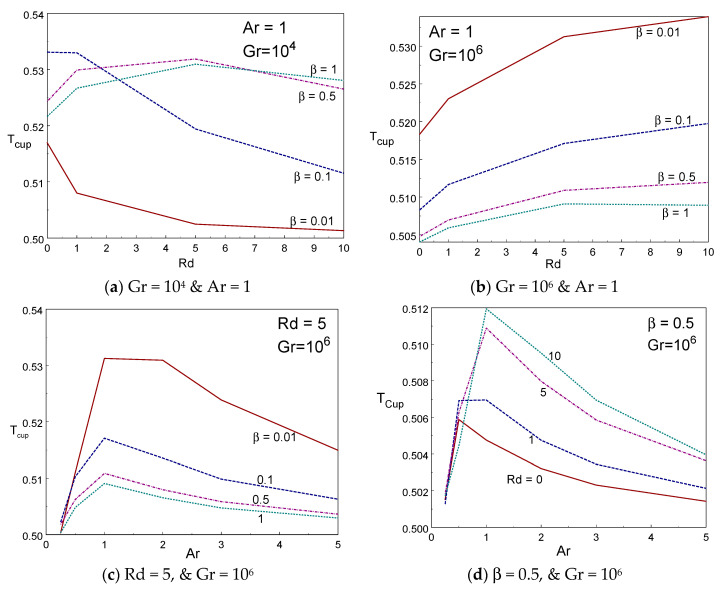
T_cup_ vs. radiation (**a**,**b**) and Ar (**c**,**d**) for different values of β and Rd.

**Figure 11:**
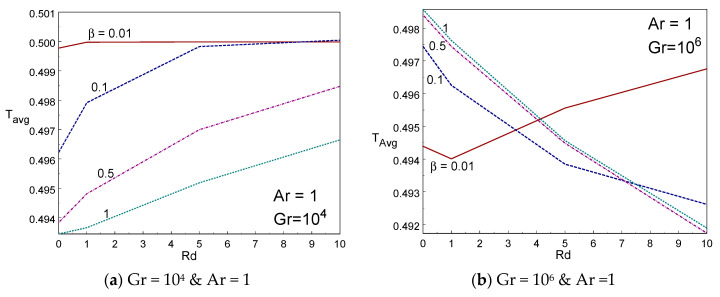

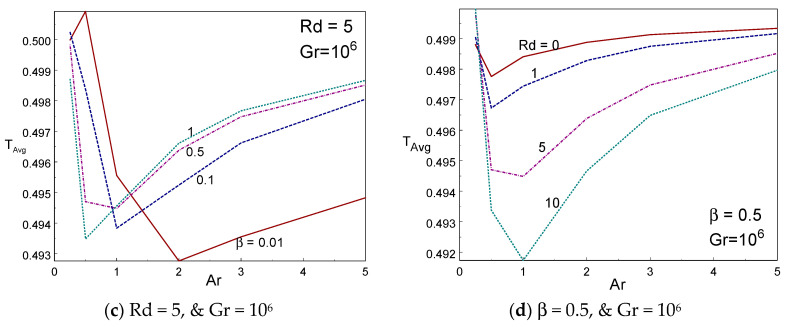
T_Avg_ vs radiation (**a**,**b**) and Ar (**c**,**d**) for different values of β and Rd.

Figure 10a–d depicted the impact of cup mixing temperature inside the box with various values of β, Rd, and Ar. The cup mixing temperature enhances while reducing the Casson fluid parameter for Gr = 10^6^. However, the opposite trend is found for Gr = 10^4^ in Figure 10a. The cup mixing temperature augments when increasing the thermal radiation for Gr = 10^6^, however, it declines when increasing the radiation for low values of Gr and low values of β (≤0.1). It is seen that the cup mixing temperature behaves nonlinearly with aspect ratio. That is, it is increasing rapidly initially and then declines gradually on raising the size of the box (aspect ratio). Figure 11a–d portrayed the impression of average temperature inside the box with various values of β, Rd, and Ar. The average temperature enhances while reducing the Casson fluid parameter. The average temperature augments when increasing the thermal radiation for Gr = 10^4^ for all values of Casson fluid parameter, however, it declines when increasing the radiation for higher values of Gr except β = 0.01. It is seen that the average temperature behaves nonlinearly with aspect ratio. That is, it is declining rapidly first (until Ar = 1) and then rises gradually on raising the aspect ratio of the box. The tall and slender cavities behave in different manner.

**Figure 12:**
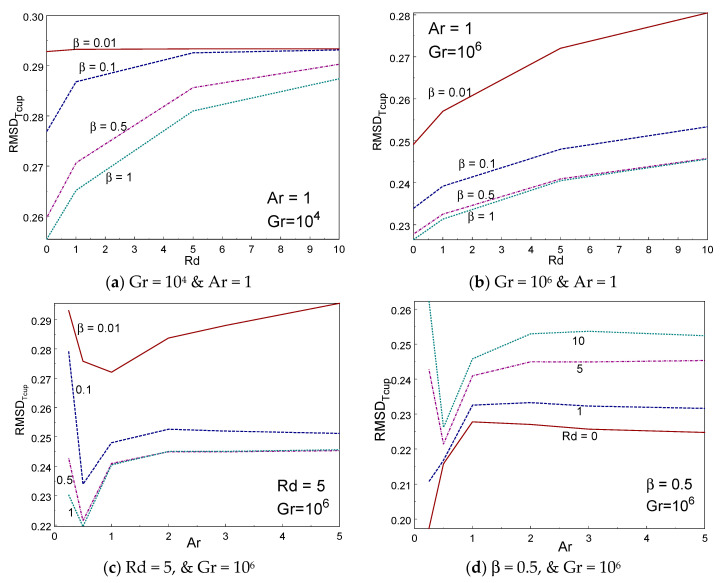
RMSD_Tcup_ vs. radiation (**a**,**b**) and Ar (**c**,**d**) for different values of β and Rd.

**Figure 13:**
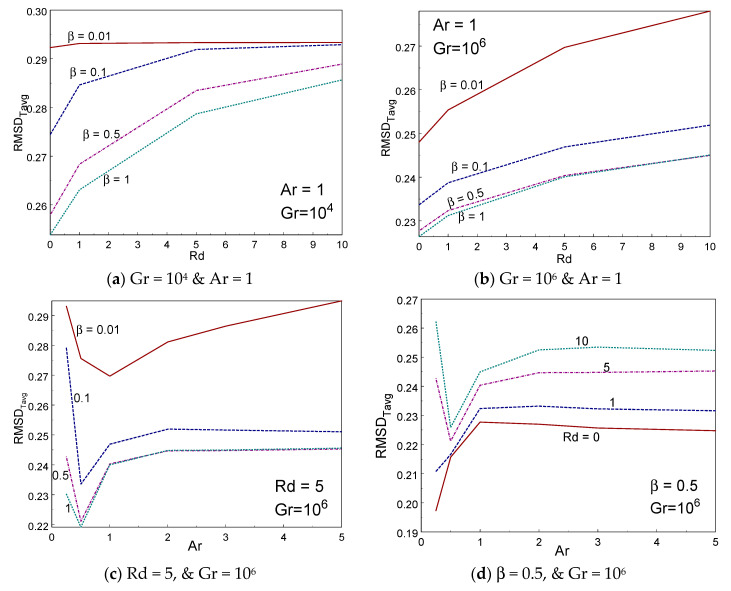
RMSD_Tavg_ vs radiation (**a**,**b**) and Ar (**c**,**d**) for different values of β and Rd.

Figure 12a–d depicted the influence of RMSD_Tcup_ inside the box with various values of β, Rd, and Ar. The RMSD_Tcup_ enhances while reducing the Casson fluid parameter. The RMSD_Tcup_ augments by increasing the thermal radiation for all values of Casson fluid parameter and Gr. It is perceived that the RMSD_Tcup_ temperature performs nonlinearly with aspect ratio. RMSD_Tcup_ declines rapidly first (until Ar = 1/2) for some cases and then rises gradually on raising the aspect ratio of the box. Figure 13a–d demonstrated the impact of RMSD_Tavg_ inside the box with various values of β, Rd, and Ar. The RMSD_Tavg_ augments while the Casson fluid parameter falls. The RMSD_Tavg_ enhances by rising the thermal radiation for all values of Casson fluid parameter and Gr. It is perceived that the RMSD_Tavg_ achieves nonlinear fashion with aspect ratio. RMSD_Tavg_ declines rapidly first (until Ar = 0.5) for some cases and then rises gradually on raising the aspect ratio of the box.

## 7. Conclusions

The impact of aspect ratio and entropy generation analysis on buoyant convective flow of Casson liquid in a rectangular box has been performed numerically. The following conclusions are arrived from this study:

○ Strong thermal layers at boundary are formed along the thermal walls.
○ Thermal stratification found for higher values of β (= 1) for all values of radiation parameter.
○ Skin friction develops with aspect ratio, thermal radiation, and Casson fluid parameter.
○ The kinetic energy enhances with aspect ratio, thermal radiation, and Casson fluid parameter.
○ Averaged heat transfer enhances with thermal radiation and Casson fluid parameter. However, it increases first and then declines when growing the aspect ratio of the box.
○ The Bejan number enhances with Casson fluid parameter and declines with Ar.
○ The cup mixing and average temperature behaves in a nonlinear fashion with aspect ratio of the box.
○ The RMSD_Tavg_ augments while the Casson fluid parameter falls and it enhances by rising the thermal radiation.
○ The RMSD_Tcup_ enhances while reducing the Casson fluid parameter and it augments by growing the thermal radiation.

## Figures and Tables

**Table 1:** Comparison of $\bar{Nu}$ for a square box with Pr = 0.71, Rd = 0, β=∞.

	$\bar{Nu}$		
Ra	Ho et al. [33]	Fusegi et al. [34]	Present
10^3^	1.118	1.106	1.103
10^4^	2.246	2.302	2.292
10^5^	4.522	4.646	4.628
10^6^	8.825	9.012	8.935
